# Fabrication of docetaxel surfaced Fe_3_O_4_ magnetite nanoparticles and their cytotoxicity on 4 T1 breast cancer cells

**DOI:** 10.1186/2008-2231-20-15

**Published:** 2012-08-30

**Authors:** MH Yazdi, Z Niazzadeh Najafi, MR Khorramizadeh, M Amini, AR Shahverdi

**Affiliations:** 1 Department of Pharmaceutical Biotechnology and Biotechnology Research Center, Faculty of Pharmacy, Tehran University of Medical Sciences, Tehran, Iran; 2 Department of Medical Biotechnology, School of Advanced Medical Technologies, Tehran University of Medical Sciences, Tehran, Iran; 3 Drug Design and Development Research Center, Faculty of Pharmacy, Tehran University of Medical Sciences, Tehran, Iran

**Keywords:** Docetaxel, Fe_3_O_4_ magnetite nanoparticles, Antiproliferative effect, 4 T1 breast cancer cells

## Abstract

**Background:**

In the recent years, there is an increasing attention to the using of Fe_3_O_4_ magnetite nanoparticles (MNPs) as drug delivery systems. Application of this nanoparticles could profit advantages of nanomedicine to enhance biological activity of pharmaceutical ingredients.

**Methods:**

Fe_3_O_4_ MNPs were synthesised by a chemical method and characterized by transmission electron microscopy and energy-dispersive spectroscopy techniques. In the next step, docetaxel-coated Fe_3_O_4_ MNPs were prepared, using percipitation method. The surface chemistry of docetaxel-coated Fe_3_O_4_ MNPs as well as their thermal decomposition characteristics were examined using fourier transform infrared spectroscopy and thermogravimetric analyzer equipment, respectively. The cytotoxicity assay was conducted on 4 T1 breast cancer carsinoma by MTT assay to evaluate the possible *in vitro* antiproliferative effects of docetaxel-coated Fe_3_O_4_ MNPs.

**Results:**

During precipitation process, docetaxel molecules were precipitated on the surface of Fe_3_O_4_ MNPs by the ratio of 3:100 w/w which indicates that each milligram of coated Fe_3_O_4_ MNPs averagely contained 30 μg pure docetaxel compound. Docetaxel showed aniproliferative effects against mentioned cell line. The higestest concentartion of docetaxel (80 μg/ml) caused about 80% cell death. However, the results demostarted that much lower amounts of docetaxel will be needed in combination of Fe_3_O_4_ MNPs to produce the potent antiproliferative effect compared to docetaxel alone. Dose response cytotoxicity assay of docetaxel-coated Fe_3_O_4_ MNPs against 4 T1 breast cancer cells showed that lower amount of docetaxel (0.6 μg/ml) can exhibit higher cytotoxic effect against this cancer cell line (90% cell death).

## Introduction

Today, side effects of anti cancer drugs are still considered as a major problem in chemotherapy of cancer diseases ([1]). In the recent years, new drug delivery systems have been developed to reduce the side effects of these drugs ([2-4]). These systems mainly include nanotubes ([5]), liposomes ([6]), dendrimers ([7]) and nanoparticles ([8]). The potential biomedical applications of Fe_3_O_4_ magnetite nanoparticles (MNPs) are well discussed in the literature ([9]). Due to their magnetic properties, these nanomaretials have recieved particular attention as possible drug carriers ([10]). The conjugation of chemical or natural anticancer drugs to Fe_3_O_4_ magnetite nanoparticles (MNPs) can lead to increse the uptake of anticancer agents by targeted tumor and improve their therapeutic index ([11]). For example, a significant difference in antiproliferation effect of a natural product, umbelliprenin, and its Fe_3_O_4_ MNPs-conjugated form was observed *in vitro* model ([12]).

**Figure 1:**
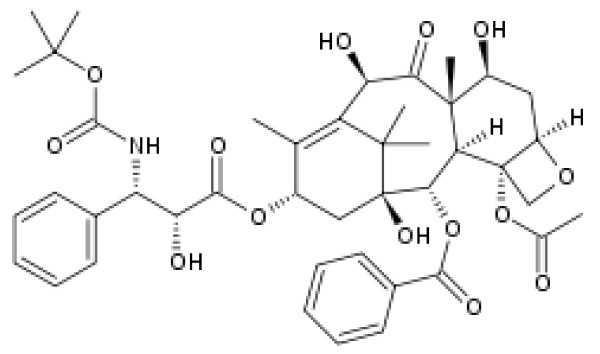
The structure of docetaxel.

Docetaxel (Figure 1) is an insoluble semi-synthetic analogue of Taxol. Docetaxel leads to a significant decrease in free tubulin, required for microtubule formation, and results in inhibition of mitotic cell division between metaphase and anaphase, preventing further cancer cell progeny ([13]). In this study, docetaxel-coated Fe_3_O_4_ MNPs have been fabricated to determine the antiproliferative effect of this anticancer agent in combination with Fe_3_O_4_ MNPs against 4 T1 breast cancer cell line. The main hypothesis is evaluation of *in vitro* cytotoxicity of docetaxel in combination of Fe3O4 MNPs on 4 T1 breast cancer cells compared to the cytotoxicity of docetaxel alone.

## Materials and methods

### Synthesis of Fe_3_O_4_ magnetic nanoparticles and their characterization

A previously reported method was used for synthesis of Fe_3_O_4_ MNPs ([14]). Briefly, deionized water (200 ml) was deoxygenated by bubbling nitrogen gas for 1.5 h, then 50 ml of NH_4_OH (1 M) was added and the mixture was stirred with mechanical agitation at 1000 rpm. Then, amounts of 6.76 g FeCl_3_.6H_2_O (2.5 mmol) and 4.97 g FeCl_2_.4H_2_O (2.5 mmol) were separately dissolved in 50 ml distilled water to make 0.5 M solutions. Subsequently, amounts of 10 ml ferrous chloride and 20 ml ferric chloride solutions were added to the ammonium hydroxide solution and the reaction mixture was stirred for 2.5 min at 1000 rpm. At this stage, a black precipitate formed which was washed four times with deionized water. Finally, using a magnet, the prepared Fe_3_O_4_ MNPs were separated from the solution. Freshly prepared Fe_3_O_4_ MNPs were characterized by transmission electron microscopy (model EM 208 Philips) and energy-dispersive spectroscopy (EDS).

### Preparation of docetaxel-coated Fe_3_O_4_ nanoparticles and their surface characterization

**Figure 2:**
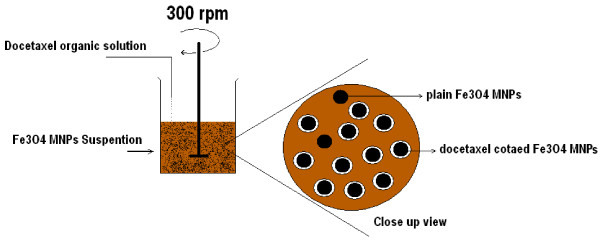
**Schematic presentation of the simple fabrication of docetaxel coated Fe**_**3**_**O**_**4 **_**MNPs used during study.**

In order to coat docetaxel on the surface of Fe_3_O_4_ MNPs, a simple precipitation method (Figure 2) was used as follows: A 2 mg/ml dichloromethane solution of docetaxel (Cipla, Mumbai, India) was prepared and reserved in a sealed container. Fe_3_O_4_ MNPs (100 mg) were dispersed in 5 ml distilled water. Thereafter, amount of 5 ml dichloromethane solution of docetaxel was added drop-by-drop to the suspension of Fe_3_O_4_ MNPs over a 15 min period under continuously stirring (300 rpm) condition, and using a laboratory magnetic stirrer. The resulting mixture was maintained in a static regime at 45°C for 24 hours. Docetaxel-coated Fe_3_O_4_ MNPs was separated from the solution by a magnet. Finally, the capped nanoparticles were washed three times with distilled water. A stock suspension of Fe_3_O_4_ MNPs and docetaxel-coated Fe_3_O_4_ MNPs were prepared (2 mg/ml) and used for antiproliferative bioassay. The surface chemistry of the docetaxel-coated Fe_3_O_4_ MNPs was studied using Fourier transform infrared spectroscopy (Nicolet Magna 550). Also thermal decomposition characteristics of docetaxel-coated Fe_3_O_4_ MNPs were determined using Thermogravimetric Analyzer *TGA* equipment as well.

### Cell culture

The 4 T1 breast cancer cell line was obtained from the National Cell Bank of Iran (NCBI), Pasteur Institute of Iran, Tehran (Iran). The cells were cultured in a RPMI-1640 medium with 10% fetal calf serum, 100 u/ml penicillin and 100 μg/ml of streptomycin in 5% CO_2_ at 37°C for one week and then used for cell proliferation assay.

### Cell proliferation assay

Thiazolyl blue tetrazolium bromide assay (MTT) was performed to evaluate cell proliferation where 2 × 10^4^ cells were seeded in 96 well plates and incubated with 0, 10, 20, 40 and 80 μg/ml of docetaxel-Fe_3_O_4_ MNPs, bared Fe_3_O_4_ MNPs or docetaxel alone for 24 hours. Then, 15 μl MTT (0.5 mg/ml) was added to each well, and the plates were incubated for 2 hrs. Then, the medium was replaced with 200 μl of DMSO and the absorbance was recorded at 570 nm ([12]). Each experiment was repeated three times and MTT assay was performed in 3 replicates for each experiments. The percentage of inhibition rate (IR) was measured using the following equation:

(1) $$\text{IR}\;\left(\%\right)\;=\;\left[1\;-\;\text{ODexp}/\text{ODcon}\right]\;\times \;100$$

Where ODexp and ODcon were optical densities of treated and untreated cells, respectively. Finally, The percentage-inhibition curve was obtained by plotting “the percentage of inhibition” (i.e.[absorbance of test wells/absorbance of control wells]/100) against the concentration of cytotoxic compounds, compared to control(i.e. untreated) cells.

## Results

### Preparation and characterization of Fe_3_O_4_ MNPs

**Figure 3:**
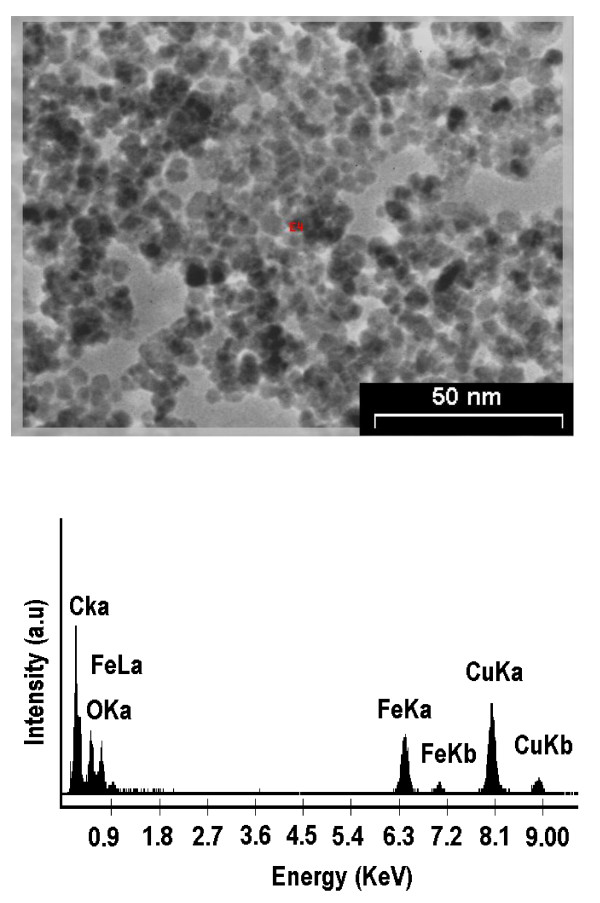
**TEM image of chemically-synthesized Fe**_**3**_**O**_**4 **_**MNPs (upper illustration) indicate that that Fe**_**3**_**O**_**4 **_**MNPs are well below 20 nm in size.** The magnetic forces between iron oxide particles -fortified by high surface area of nanoparticles- caused agglomeration among MNPs. Lower illustration shows energy dispersive spectrum (EDS) of Fe_3_O_4_ MNPs which confirms the presence of Fe and O atoms and the existence of iron-oxide nanoparticles. Additional peaks, of copper and carbon elements are attributed to the grid used for TEM imaging.

As mentioned earlier, Fe_3_O_4_ MNPs were chemically synthesized and their shape as well as size properties were confirmed by transmission electron microscopy. Upper illustration in Figure 3 demonstrates a representative TEM image of synthesized Fe_3_O_4_ MNPs. TEM image shows that the particles range in size below than 20 nm and possess an average size of 10 nm. The lower illustration in Figure 3 depicts the energy dispersive spectrum of Fe_3_O_4_ MNPs revealed the presence of Fe and O elements peaks. These EDS peaks confirm the existence of iron-oxide nanoparticles. It should be mentioned that additional peaks, belonging to copper and carbon element are attributed to the grid used for TEM imaging.

### Preparation and surface characterization of docetaxel coated Fe_3_O_4_ MNPs

**Figure 4:**
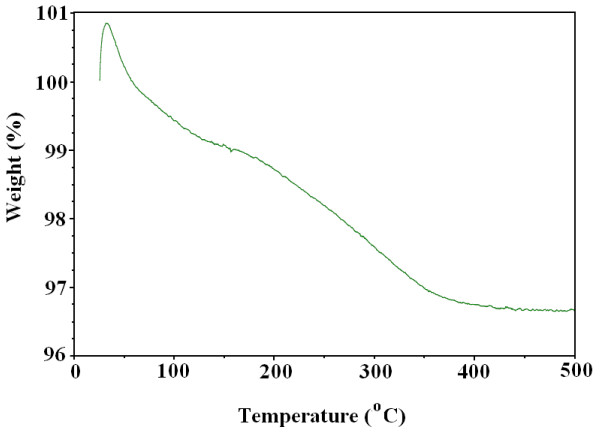
**Weight loss of docetaxel-coated Fe**_**3**_**O**_**4 **_**MNPs as a function of temperature which was determined using thermogravimetric method.** This weight loss in the TGA curve indicated carbon oxidation which proved the presence of organic moieties in the sample, thereby confirming that Fe_3_O_4_ MNPs are truly coated with docetaxel molecules.

After drop-by-drop addition of the organic solution of docetaxel to Fe_3_O_4_ MNPs slurry, docetaxel molecules precipitated on the surface of the Fe_3_O_4_ nanoparticles by the ratio of about 3/100 w/w (it means that 1000 μg of coated Fe_3_O_4_ MNPs nanoparticles contained almost 30 μg docetaxel). This ratio was simply determined gravimetrically by weighing the coated Fe_3_O_4_ MNPs before and after it was washed with chloroform as solvent, for three times as well as the results obtained from thermogravimetric assay. Figure 4 demonstrates weight loss of docetaxel-coated Fe_3_O_4_ MNPs as a function of temperature which was determined using this method. This weight loss (%3) in the TGA curve indicated carbon oxidation which proved the presence of organic moieties in sample, thereby confirming that Fe_3_O_4_ MNPs are truly coated with docetaxel molecules. The associated FTIR spectroscopic data of docetaxel-coated Fe_3_O_4_ MNPs, docetaxel-free Fe_3_O_4_ MNPs and docetaxel alone, are outlined in Table 1; which further confirmed the presence of docetaxel on the surface of synthesized Fe_3_O_4_ MNPs. In terms of chemical bonding, a main interaction is expected to occur between Fe_3_O_4_ MNPs and docetaxel: electrostatic bounds may form between carbonyl oxygen and hydroxyl groups of docetaxel with Fe chelating agent in Fe_3_O_4_ crystal (Table 1). This interaction lead to disappear of hydroxyl bands centered at 3487 and 3377 cm^-1^ in FTIR spectrum of docetaxel-coated Fe_3_O_4_ MNPs. It is possible that these interactions make docetaxel -coated Fe_3_O_4_ MNPs a suitable carrier with bipolar properties which penetrates into both hydrophilic and hydrophobic (i.e. cell membrane) fluids at the same time.

**Table 1:** **The FTIR spectroscopy data of Fe**_**3**_**O**_**4**_**MNPs, docetaxel and docetaxel coated Fe**_**3**_**O**_**4 **_**MNPs**

**Sample**	**Main peaks wave numbers (cm**^**-1**^**)**
Fe_3_O_4_ MNPs	584
docetaxel-coated Fe_3_O_4_ MNPs	2922, 2927, 1736, 1246, 1161, 1065
docetaxel alone	3487, 3377, 2937, 1735, 1712, 1246, 1169, 1072

Underlined data shows common corresponding peaks in FTIR spectra of docetaxel-coated Fe_3_O_4_ MNPs and docetaxel.

### Antiproliferative effects

**Figure 5:**
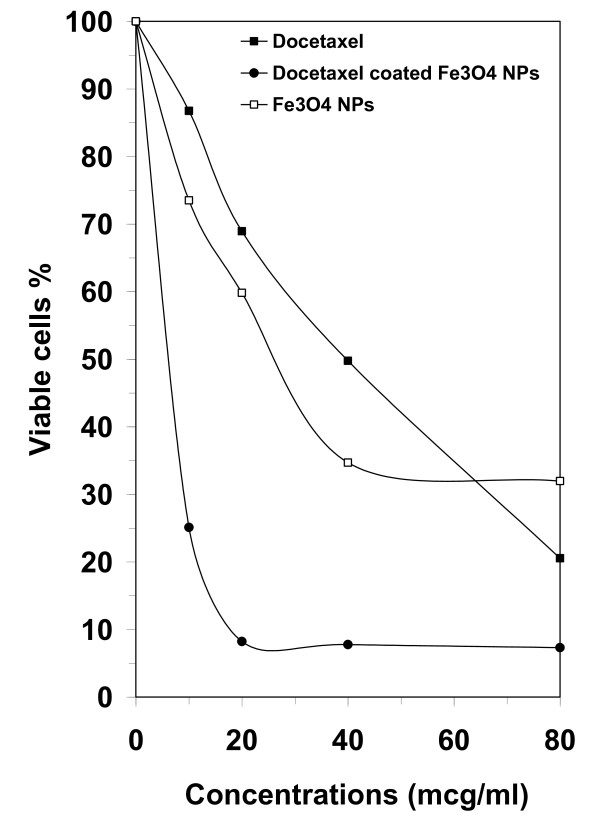
**Antiproliferative effect of Fe**_**3**_**O**_**4 **_**MNPs, docetaxel and docetaxel-coated Fe**_**3**_**O**_**4 **_**MNPs against 4 T1 breast cancer.**

Antiproliferative effects of docetaxel-coated Fe_3_O_4_ MNPs, docetaxel alone, and Fe_3_O_4_ MNPs alone were *in vitro* evaluated at different concentrations on 4 T1 breast cancer cells. Cytotoxicity analysis of samples suggested a direct dose–response relationship, in which cell proliferation decreased in higher concentrations (Figure 5). Samples were found to have different cytotoxicities on 4 T1 breast cancer cells, with docetaxel-coated nanoparticles being the most potent. The antiproliferative effect of test compounds increased in the following order: docetaxel < Fe_3_O_4_ MNPs < docetaxel-coated Fe_3_O_4_ MNPs. Moreover, the necessary concentrations to cause 50% cell death (IC_50_) were more than 30 and 40 (μg/ml) in cells treated with Fe_3_O_4_ MNPs alone and docetaxel, respectively. Docetaxel-coated Fe_3_O_4_ MNPs could cause the same effect at 10 μg/ml concentration. In other words, the combination of docetaxel and Fe_3_O_4_ MNPs exhibited a much more potent antiproliferative activity than docetaxel and Fe_3_O_4_ MNPs alone. As clear in Figure 5, docetaxel at concentrations of 20 and 30 (μg/ml), moderately reduced the proportion of viable cells to 70% and 50%, respectively. The cytotoxicity curve for Fe_3_O_4_ MNPs alone followed almost similar pattern, though sharper. The percentage of viable cell number sharply decreased from 87% at the concentration of 10 μg/ml to 35% at 40 μg/ml concentration. In contrast, however, only the low concentration of 10 μg/ml docetaxel-coated Fe_3_O_4_ MNPs (equivalent to the concentration of approximately 0.3 μg/ml pure docetaxel), drastically reduced the proportion of viable cells by more than 70%. Also a considerable cytotoxicity (about 90%) was observed for 20 μg/ml docetaxel-coated Fe_3_O_4_ MNPs against 4 T1 breast cancer cells. In higher concentrations (>20 μg/ml), this proportion declined steadily, until it reached the remarkably-low amount of about 7% at 80 μg/ml concentration.

## Discussion

Literature review shows that there are numerous reports on cytotoxicity of docetaxel in combination of different carrier ([15,16]). However, the efficiency of this compound in conjugation with Fe_3_O_4_ MNPs has not been yet investigated. Here, the antiproliferative effect of docetaxel enhanced in tumor cells when it was coated on the surface of Fe_3_O_4_ MNPs has been reported. As mentioned earlier, Fe_3_O_4_ MNPs, docetaxel and docetaxel-coated Fe_3_O_4_ MNPs, all showed antiproliferative effects on 4 T1 breast cancer cells, increasing in the order: docetaxel < Fe_3_O_4_ MNPs < docetaxel-coated Fe_3_O_4_ MNPs. It is noticeable that based on current study, docetaxel has considerable antiproliferative effect on 4 T1 breast cancer cells in concentrations of not below 10 μg/ml. However, obtained results demonstrated that when coated on Fe_3_O_4_ MNPs surface, docetaxel have antiproliferative effect, even at the low concentration of 0.3 μg/ml. This literally means that much lower amounts of docetaxel will be needed to produce the same antiproliferative effect as docetaxel alone. As described in above, during precipitation process, docetaxel molecules were precipitated on the surface of Fe_3_O_4_ MNPs by the ratio of 3/100 w/w which indicates that each milligram of coated Fe_3_O_4_ MNPs averagely contained 30 μg pure docetaxel compound. Therefore, it is deducible from Figure 5, that the concentration of 80 μg/ml docetaxel caused 79% cell death. However, when combined with Fe_3_O_4_ MNPs, the average concentration of 0.6 μg/ml docetaxel (which is equivalent to the concentration of about 20 μg/ml docetaxel- Fe_3_O_4_ MNPs) caused more than 90% cell death. Thus, it is concluded that by coating docetaxel on the surface of Fe_3_O_4_ MNPs, the required amount of docetaxel for showing a potent antiproliferative effects against 4 T1 breast cancer cells could be considerably lowered (more than 100 fold). As mentioned before, many pharmaceutical compounds have low solubility in biological fluids, which reduces their efficiency in *in vivo* models. Therefore, it is hypothesized that, Fe_3_O_4_ MNPs may be considered as efficient carriers for these insoluble compounds.

## Conclusion

In this paper, the antiproliferative effect of docetaxel (IC_50_ =40 μg/ml) has been reported and the results revealed that the combination of Fe_3_O_4_ MNPs and docetaxel is significantly more cytotoxic (IC_50_ =10 μg/ml) than docetaxel alone. In conclusion, this result is of particular value since it demonstrates that by using metallic nanoparticles in combination therapy, lower amounts of these materials might be required, resulting in reduction of the potential hazards of docetaxel usage. Further investigations are needed to determine the antiproliferative effect of docetaxel-coated Fe_3_O_4_ MNPs in other cell lines. Moreover, studies on cytotoxic effects of docetaxel-coated Fe_3_O_4_ MNPs in animal models will be of great value.

## Competing interests

The authors declare that they have no competing interest.

## Authors’ contibutions

MHY: Collaboration in proliferation assays. ZNN: Collaboration in fabrication of nanoparticles. MRK: Commented on cytotoxic assays. MA: Commented on surface chemistry analysis of the nanoparticles. ARS: Design of the project. All authors read and approved the final manuscript.
